# Empyema caused by Streptococcus constellatus in a patient infected with HIV: a case report and literature review

**DOI:** 10.1186/s12981-023-00587-z

**Published:** 2024-01-03

**Authors:** Hong-Hong Yang, Mei Li, Qing Yu, Qian Liu, Min Liu

**Affiliations:** Division of Infectious Diseases, Chongqing Public Health Medical Center, 109 Baoyu Road, Shapingba District, Chongqing, 400036 China

**Keywords:** Streptococcus constellatus, HIV, Empyema, Culture, Treatment

## Abstract

**Background:**

Empyema caused by Streptococcus constellatus (S. constellatus) is rare in patients with HIV. To analyze the clinical data of a patient living with HIV (PLHIV), who got empyema caused by S. constellatus, investigating the diagnosis and treatment of this disease through literature review to improve the clinical understanding of this disease.

**Case presentation:**

We have reported here a 58-year-old male PLHIV with cough, wheezing, and fever for 20 days. He has a history type 2 diabetes, alcohol abuse, and a teeth extracted. Chest computed tomography revealed multiple encapsulated pleural effusions, pneumatosis, and partial compressive atelectasis in the right lung. Submission of pleural efusions timely, and then cultures revealed S. constellatus. After comprehensive treatment, including antibiotics, closed pleural drainage, and intrapleural injection of urokinase, the pleural efusion was absorbed, and chest computed tomography also confirmed the improvement.

**Conclusions:**

S. constellatus should not be neglected as a pus pathogen in patients with HIV. comprehensive treatment is important for empyema of S. constellatus.

## Introduction

Empyema is characterized by pus or bacteria in the pleural space, which is an important clinical problem with high morbidity and mortality rates nowadays [1, 2]. Streptococcus constellatus (S. constellatus) belongs to a member of Streptococcus anginosus group (SAG), which is found in the normal flora of the oral cavity, nasal cavity, intestinal tract, etc. [3]. It is not usually considered a pathogen and may be underestimated. In recent years, there are gradually reported that S. constellatus induces abscesses mainly in brain, mediastina, liver, bone and soft tissues [4–9] with the improvement of detection technology. However, there are few reported about human immunodeficiency virus (HIV)/acquired immunodeficiency syndrome (AIDS) combined with S. constellatus, although it has been reported that the pathogen may cause invasive infections after entering sterile sites in the body, especially in patients with immunosuppression [10]. Here, we have reported a case of a 58-year-old patient living with HIV (PLHIV) who got empyema caused by S. constellatus, then cured successfully by antibiotic, closed thoracic drainage and intrapleural injection of urokinase.

## Case report

A 58-year-old male patient was admitted to our department due to cough, wheezing, and fever for 20 days. The patient was confirmed to be HIV positive two month ago, the baseline CD4 + T-cell count and HIV-RNA level were unknown, antiviral therapy (ART) has been given with BIC/FTC/TAF. His past medical history included type 2 diabetes, alcohol abuse, and a 30-year pack history of tobacco smoking. In addition, the patient’s oral hygiene was not good, who had a history of gum swelling and pain, and one teeth had been extracted three month ago.

20 days before admission, the patient begun to paroxysmal cough and expectoration. Accompanying with shortness of breath mainly after exercise, and fever mainly in the afternoon and night, with a maximum body temperature of 39℃. Moreover, he had no chest tightness, chest pain, hemoptysis, palpitations, or other discomforts. So he was admitted to a local hospital, and chest computed tomography (CT) revealed infectious lesions in both lungs and a amount of right-sided pleural effusion. Closed thoracic drainage was performed to drain approximately 2000 ml of milky white pleural fluid, and received antibiotic therapy (specific drugs were unknown), however, symptomatic improvement was not noted. The patient was subsequently transferred to our hospital for further treatment.

**Fig. 1:**
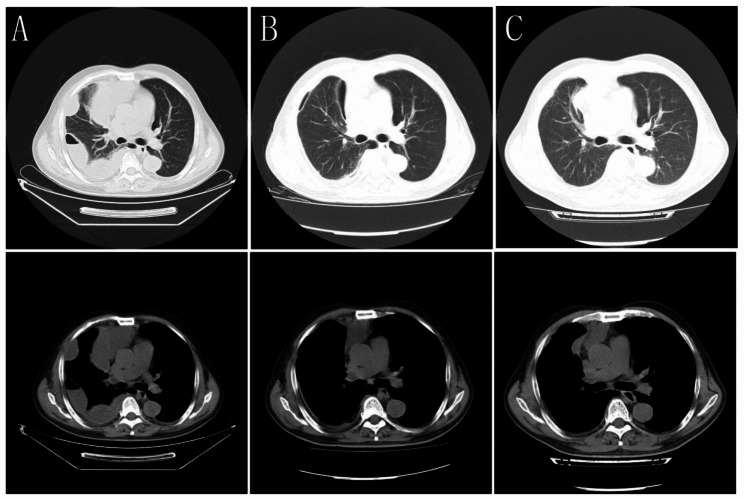
Chest CT. (*A*) before treatment, revealed multiple encapsulated pleural effusions, pneumatosis, and pleural thickening on the right side, partial compressive atelectasis in the right lung; a small number of scattered infection foci in the right lung; multiple small bullae in both lungs. (*B*) Twelve days after treatment. (*C*) Four weeks after treatment, (*B*) and (*C*) indicate that pleural effusion were improved

On admission, his temperature was 37.0 ℃, pulse rate 116 beats/min, respiratory rate 30 breaths/min, blood pressure 148/82–mmHg, and oxygen saturation 94% on room air. Oral examination showed calculus and a tooth defect. No lymphadenopathy was detected. Pulmonary auscultation found decreased breath sounds on right lower fields. No abnormalities were detected upon cardiac and abdominal physical examination, Moderate edema of both lower limbs. The results of his laboratory examination were as follows: arterial blood gas revealed PH 7.34 (normal range: 7.35–7.45), oxygen pressure (PaO2) 58–mmHg (normal range: 83–108–mmHg) and oxygenation index 276–mmHg (normal range: 400–500 mmHg), white blood cell (WBC) count 14.84 × 10^9/L (normal range, 3.5–9.5 × 10^9/L), neutrophil ratio 89.90% (normal range, 40–75%). procalcitonin (PCT) 2.347 ng/ml (normal range, 0–0.05 ng/ml), C-reactive protein (CRP) 309.55 mg/L (normal range, 0-4 mg/L) with a erythrocyte sedimentation rate (ESR) of 66 mm/h (normal range, 0–20 mm/h), serum albumin 27.9 g/L (normal range, 40-55 g/L) and his fasting blood glucose was 12.98mmol/L (normal range, 3.9–6.1 mmol/L). CD4 + T-cell count 61 cells/µl, CD4/CD8: 0.46; HIV viral loads: 1.02E + 02 (copes/ml). Chest enhancement CT revealed multiple encapsulated pleural effusions, pneumatosis, and pleural thickening on the right side, partial compressive atelectasis in the right lung; a small number of scattered infection foci in the right lung; multiple small bullae in both lungs (Fig. 1). Subsequently, thoracocentesis was performed and a chest tube was introduced. Milky white pleural fluid was aspirated, laboratory analysis showed WBC count 230,600/mL, polymorphonuclear neutrophil count 93%, lactate dehydrogenase 7230IU/L (normal range, 109-245U/L), glucose 1.4 mg/dL (normal range, 2.4-4.5 mg/dL), protein level 8.0 g/L (normal range, 0–30 g/L), and adenosine deaminase 352.5 IU/L (normal range, 0–24 IU/L). Pleural fluid smear, gram stain, acid-fast bacilli culture and smear, and cytology were all negative. Electrocardiogram (ECG), abdominal ultrasound and fiberbronchoscopy were no abnormal changes. Culture specimens (including blood, sputum, pleural water and bronchoalveolar lavage fluid (BALF)) are retained before antibiotic.

The primary diagnosis were 1. empyema, 2. sepsis (according to SOFA score) [11], 3. AIDS, 4. type 2 diabetes, 5. hypoalbuminemia. It is vital to administer empirical antibiotics before bacterial culture results, especially in patients with severe infections. For empyema, the British Thoracic Society (BTS) and American Association for Thoracic Surgery suggest broad-spectrum antibiotics with Gram-positive, Gram-negative and anaerobic cover until culture and sensitivities are available [12, 13], so intravenous empiric meropenem (1 g once every 8 h) was commenced. He also continued to receive BIC/FTC/TAF for anti-human immunodeficiency virus (anti-HIV) treatment and metformin combined with glimepiride for controlling blood sugar. Meanwhile, the pleural effusion was drained continuously through the chest tube, and received repeated washout with urokinase (100000U/d) to avoid insufficient drainage.

Culture tests of the pleural effusion identified the presence of S constellatus on the fourth day of hospitalization, which was sensitive to penicillin, levofloxacin, ceftriaxone, linezolid and vancomycin, but resistant to tetracycline and clindamycin (Table 1), whereas S. constellatus was not detected in culture tests of the patient’s blood, sputum and BALF. After 3 days of treatment, body temperature returns to normal (fever on day 1 and day 2) and laboratory test results (WBC count 6.84 × 10^9/L, neutrophil ratio 78.90%, PCT 0.556 ng/ml, CRP 114.99 mg/L, PCT 0.556 ng/ml) showed improvement, antibiotic therapy was changed to intravenous amoxicillin clavulanate potassium (1.2 g once every 8 h) and metronidazole (0.2 g once every 8 h) according to the antibiotic susceptibility test results. On the 12th day of admission, the close thoracic drainage tube was removed, and the patient claimed gradually resolved symptoms under antibiotic treatment and effusion drainage, and then he was discharged for economic reasons. He continue to take a two-weeks oral amoxicillin clavulanate potassium tablet (1.2 g once every 8 h) after discharge. One month after treatment, repeat chest CT showed resolution of empyema (Fig. 1).

**Table 1:** Drug sensitivity to S. constellatus

Drug	Pleural effusion culture	
	Sensitivity	MIC (ug/ml)
Penicillin G	Sensitive	≤ 0.06
Ampicillin	Sensitive	≤ 0.25
Vancomycin	Sensitive	≤ 1
Ceftriaxone	Sensitive	≤ 0.5
Tetracycline	Resistant	= 32
Linezolid	Sensitive	≤ 2
Levofloxacin	Sensitive	= 2
Ciprofloxacin	Sensitive	= 1
Erythromycin	Resistant	≥ 16
Clindamycin	Resistant	≥ 8

MIC, minimum inhibitory concentration

## Discussion

S. constellatus is a conditional pathogenic bacterium and one of Streptococcus pyogenes, which can cause purulent infection in various organs of the body when the body’s resistance decreases [14–16]. In recent years, S. constellation has gradually become one of the important pathogens that cause purulent infection in the body. Empyema is caused by pathogenic bacteria invading the pleural cavity, which has increased globally. It has been reported that the common pathogenic bacteria are aerobic (Streptococcus pneumoniae, Staphylococcus aureus, Klebsiella pneumoniae, and Enterobacteriaceae), and anaerobic (Bacteroides and Peptostreptococcus spp.) [16]. By searching the English medical literature in PubMed and the Chinese literature in China National Knowledge Infrastructure, there are 29 literatures (including 12 Chinese and 17 English) of empyema caused by S. constellation in recent 20 years, but there are almost no reports of AIDS/HIV combined with S constellatus.

S. constellatus is a facultative anaerobe, a gram-positive coccus, and catalase negative. Bacterial culture is the gold standard for the diagnosis, we should pay attention to the following point. Firstly, It is easy to be missed in the microbial culture tests performed in aerobic environments, because this bacteria needs to be placed in 5% CO2 or anaerobic environments to grow [17]. At present, the wide application of metagenomics next-generation sequencing (mNGS) has dramatically improved the etiological detection rate [18], however, mNGS also has its own drawbacks, a drug sensitivity test cannot be performed to clarify the drug susceptibility compared to traditional pathogenic tests such as blood and sputum cultures, and it’s relatively expensive. Secondly, the bacterium is sensitive to most of antibiotics [19, 20], it is easy to have a negative culture result due to the overuse of antibiotics. Samples were taken immediately in the case, thus making the diagnosis clear, this once again demonstrated the importance of timely submission of specimens. Thirdly, S. constellatus observed in samples are often overlooked and erroneously considered as commensal contamination, this emphasis the importance of the communication between clinicians and laboratory technicians.

S. constellatus infection is not common, but can cause abscesses and bacteremia, which may be related to the structure of polysaccharidic capsules on these bacteria [21]. It may be induce infections in patients with alcohol abuse, chronic respiratory diseases, immunodeficient diseases, diabetes mellitus or cardiovascular diseases, etc. [10, 14]. Our patient suffered from HIV with a low level of CD4 T-lymphocytes (61 cells/µl), a history of diabetes and alcohol abuse, so he was prone to be infected by this bacterium. The origin of the S. constellatum in our patient is unclear, it seems that the odontogenic infections is the main invasion path, because the patient recently had a tooth extraction.

The sufficiently treatment for empyema is treated with thoracic drainage and antibiotics. S. constellatus is sensitive to a range of antibiotics, penicillins are the first choice, followed by erythromycin, chloramphenicol, clindamycin, ofloxacin, vancomycin, cephalosporin, and other antibiotics. It’s recommended those patients with pleural infection should early receive empirical antibiotics by using the above-mentioned drugs. It’s reported that the S. constellatus tends to co-infect with other anaerobes [16]. The presence of anaerobes can positively enhance infection of S. constellatus, and associated with a high incidence of lung abscess and mortality. The potential mechanism was that anaerobes and its metabolite inhibit the bactericidal activity of the host and stimulate the growth of S. constellatus, by impairing the function of polymorphonuclear leukocytes (PMNLs) [22, 23]. So antibiotics should against both S. constellatus and anaerobes. Although consensus or universal guidelines are lacking for S.constellatum, the recommended general duration of antimicrobial treatment for empyema is 2–6 weeks. In our case, the patient was treated with amoxicillin clavulanic acid potassium combined with metronidazole according to drug susceptibility testing, following up with amoxicillin clavulanic acid potassium after discharge for 2-week, completing a approximately 4-week course of antibiotic treatment.

Early and adequate pleural drainage is very important for the treatment of empyema. However, smooth drainage is usually not possible when in cases where pus has formed, fibrin is deposited, and septa have formed in the thoracic cavity. At this time, closed thoracic drainage combined with intrapleural injection of urokinase is recommended, which has the following advantages: less invasive, has fewer side effects, shortens the hospitalization period.

In summary, this case reported a patient living with HIV who presented with empyema caused by S constellatus, which was successfully treated with antibiotics therapy, closed thoracic drainage and intrapleural injection of fibrinolytic enzymes. S. constellatus should not be neglected as a pus pathogen in patients with HIV. In addition, we should pay attention to timely submission of traditional bacterial culture although NGS was widely used.

## Data Availability

The data that support the findings of this study are openly available.

## Abbreviations

S. constellatus: Streptococcus constellatus
PLHIV: Patient living with HIV
SAG: Streptococcus anginosus group
ART: Antiviral therapy
mNGS: Metagenomics next-generation sequencing
